# Depolymerisation of poly(lactide) under continuous flow conditions†

**DOI:** 10.1039/d4sc05891g

**Published:** 2024-11-12

**Authors:** Sophie Ellis, Antoine Buchard, Tanja Junkers

**Affiliations:** a Polymer Reaction Design Group, School of Chemistry, Monash University 17 Rainforest Walk Clayton VIC 3800 Australia tanja.junkers@monash.edu; b Department of Chemistry, Institute for Sustainability, University of Bath Claverton Down Bath BA2 7AY UK; c Department of Chemistry, University of York York YO10 5DD UK antoine.buchard@york.ac.uk

## Abstract

Poly(l-lactic acid) (PLLA) is commercially successful bio-based plastic, where end-of-life materials can undergo industrial composting. To create a circular economy, a desirable alternative to composting is chemical recycling to monomer (CRM), where direct depolymerisation to l-lactide can be achieved. CRM of PLLA is typically impeded by thermal decomposition and side reactions, due to the high ceiling temperate (*T*_c_) of PLLA in bulk (>600 °C), which preclude implementation on a large scale, and has led to the development of catalytic strategies, under vacuum or high dilution in high boiling point solvents conditions. In this study, a commercially available Sn(ii) catalyst and low boiling point solvents, at a range of temperatures and concentrations, were explored for the CRM of PLLA in a continuous flow process. The solvent THF was found to produce the best results, where up to 92% conversion of lactide could be achieved, with 92–97% selectivity for l-lactide formation at temperatures 150–170 °C. Further, inline monitoring of monomer and polymer concentrations in flow were used to determine the depolymerisation rate coefficient *k*_depo_ and the activation energy of *k*_depo_ was determined to be 129.4 kJ mol^−1^.

## Abstract 2

Poly(l-lactide) is depolymerised in continuous flow, allowing to use benign solvents such as THF to reach up to 90% depolymerisation efficiency.

## Graphical abstract

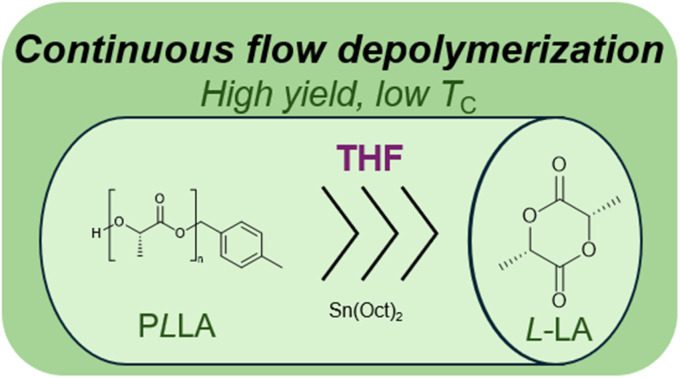

## Introduction

Poly(lactide) (PLA) is a bio-sourced thermoplastic polymer with a versatile range of applications, including packaging, 3D printing and uses in the biomedical sector.^1–4^ PLA is made up of lactic acid repeat units and can be produced directly from lactic acid or its cyclic dimer, lactide. Lactic acid is typically obtained from the fermentation of starch and has been demonstrated to have a low carbon footprint.^5^ While not readily degradable in the open environment, PLA is fully compostable under industrial conditions (norm EN13432).^6^ Other methods for dealing with end-of-life (EOL) PLA, such as mechanical recycling or incineration, ultimately lead to lower quality plastics or loss of molecular complexity resulting in economic losses.^7–9^ Therefore, an alternative approach to dealing with EOL PLA is desired, in order to achieve true circularity for its use and production.^10^ Lifecycle assessments of PLA have found that most of the process's energy input is used in production of l-LA from the plant, meaning the greatest energy intensive steps must be repeated if PLA is composted and the starch resource regrown.^11,12^ PLA can alternatively be degraded to the one of the monomer's precursors, lactic acid, or alkyl lactates.^13–15^ Whilst this creates a closed loop system, there is still a significant energy input required to transform lactic acid to l-LA, therefore, the direct chemical recycling to the (lactide) monomer (CRM) is most desirable to minimise energy inputs.^16^ Direct depolymerisation to lactide results in a monomer feedstock which can be repolymerised to the original or modified polymer without loss of quality, allowing indefinite recycling and creating a fully closed-loop cycle.

A pathway to chemical recycling of polymers is given by the thermodynamics of a polymerisation reaction. Every polymerisation reaches by definition at some temperature an equilibrium state when the enthalpy term becomes as large as the entropy part in the Gibbs free energy equation.^17^ The temperature by which this occurs is called the ceiling temperature *T*_c_. *T*_c_ is, however, not an absolute, and depends on the monomer concentration, as presence of higher monomer concentrations will favour the forward polymerisation reaction, while absence of monomer favours the reverse depolymerisation. The relationship between the equilibrium monomer concentration [*M*]_eq_ and the thermodynamic parameters is given in eqn (1).1
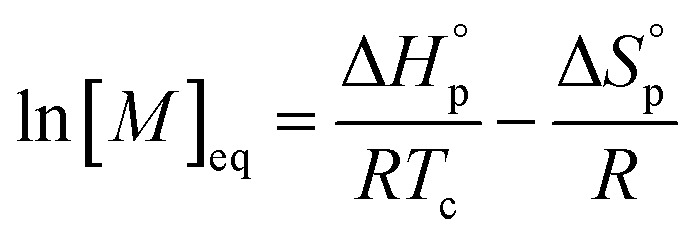


Rearrangement of eqn (1) yields the ceiling temperature.^17^2
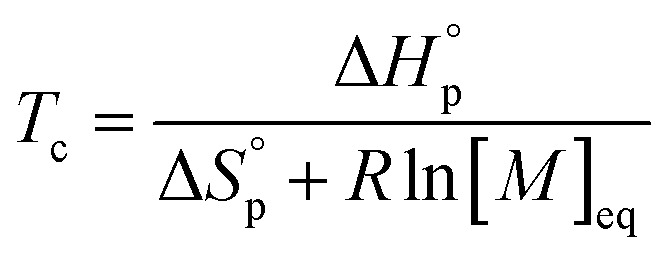


If the ceiling temperature of a given polymer is lower than the temperature of decomposition, then raising the polymer above this temperature should favour depolymerisation of the polymer to recover the initial monomer (if the polymer is in its reactive form).^17^ However, if the ceiling temperature is higher than the decomposition temperature then a mixture of products will be obtained due to competition of depolymerisation and degradation occurring simultaneously.^18^ Most CRM processes have been demonstrated with new monomers within research, but for already existing commercial plastics this has not yet been fully achieved.^19–22^l-LA CRM is rather challenging due to the high *T*_c_ of PLA when present in bulk (>600 °C).^23,24^ The elevated temperature required for this process leads to thermal degradation (*T*_d,PLLA_ ∼ 350 °C) and side reactions, including epimerisation which produces d-lactide and *meso*-lactide, and elimination reactions which can form acrylic acid.^18^

However, under vacuum and reactive distillation conditions, *T*_c_ can be lowered below the onset of thermal decomposition, and the use of catalysts such as tin(ii) 2-ethylhexanoate (Sn(Oct)_2_) and zinc(ii) chloride or zinc acetate (Zn(OAc)_2_) have also been shown to improve the selectivity towards LA.^18,25,26^ Enthaler and co-workers have thus successfully depolymerised EOL PLA in bulk under reduced pressure, using Zn(OAc)_2_ (0.4 mol%) as a catalyst and temperatures of 200–210 °C, producing 98% LA in 6 hours, with around 12% *meso*-LA content.^25^ Previous work has shown rates of depolymerisation to be dependent on the degree of polymerisation, where high molar mass PLA have slower depolymerisation rates.^27^ To overcome this issue, a recent two-step, one-pot process was designed by where high molecular weight PLA first underwent transesterification with a triol to produce a low molecular weight PLA, followed by depolymerisation to l-LA.^28^ The reaction achieved 92% conversion in ∼3 hours, with >99% l-LA selectivity (5 mbar, 0.01% Sn(Oct)_2_, 160 °C). A similar concept was applied by Byers and co-workers using a ZnCl_2_/PEG600 catalyst system.^26^

In an alternative approach, Odelius and co-workers focussed on lowering the *T*_c_ of LA through the use of solvents.^29^ Increasing the dilution of the system will decrease the *T*_c_ by lowering the absolute monomer concentration below the equilibrium concentration, and in low *T*_c_ systems simple dilution is enough to drive depolymerisation.^30,31^ However, in high *T*_c_ systems like LA dilution is not enough and the monomer–polymer–solvent interactions were found to be key for suppressing *T*_c_.^29^ They found *T*_c_ of LA to be reduced to as low as 119 °C, allowing for up to 96% recovery of LA at 140 °C (99% l-LA selectivity, DMF, 0.5 M, 10 mol% Sn(Oct)_2_). However, the solvents explored all had high boiling points (>150 °C), resulting in a low yield of LA isolation (38%).

**Fig. 1:**
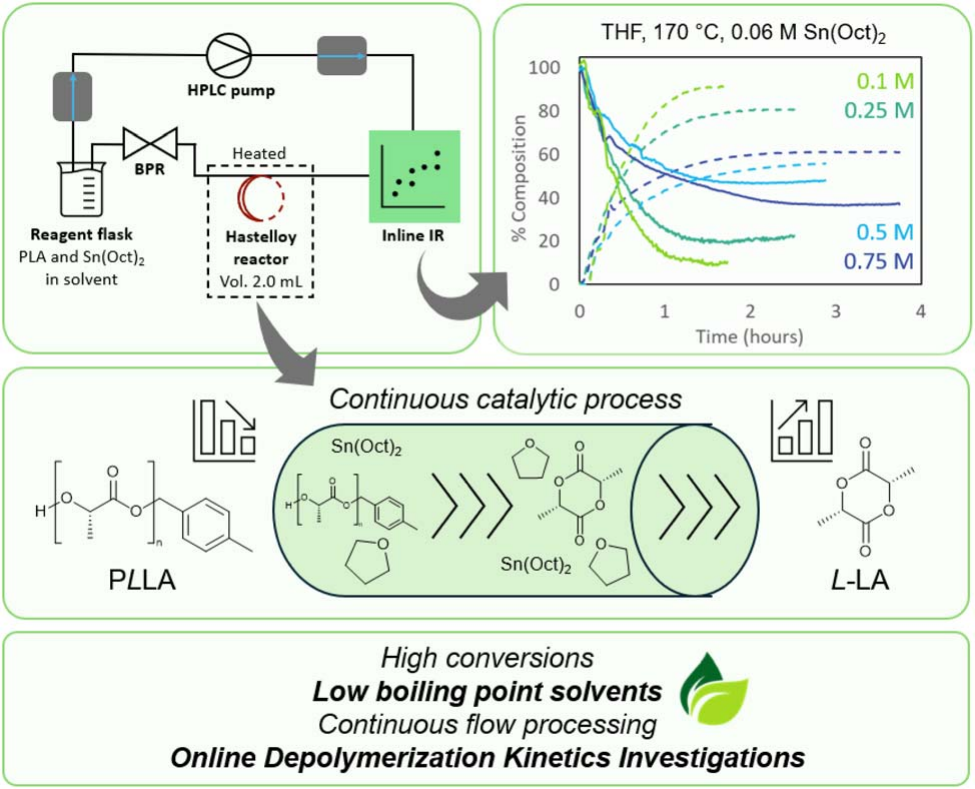
Overview of PLA depolymerisation under continuous flow conditions.

For CRM of a commercial polymer like PLA to be relevant industrially, the process needs to be scalable, the depolymerised monomer must be easily recoverable, and the process needs to be energy efficient. While energy efficiency can be reached by decreasing the ceiling temperature, effective methods of heating also present ideal solutions. Flow chemistry allows to utilise both factors for a beneficial depolymerisation process. The closed and typically slightly overpressured flow reactors allow for the use of low boiling point solvents for high temperature reactions. At the same time, flow chemistry offers excellent heat transfer capabilities. This typically results in the ability to flash heat a reaction with high temporal control, and with a narrow temperature distribution. It is this narrow distribution that classically allows to carry out flow reactions in widened process windows (usually higher temperatures) with increased yields (as side product reaction pathways are diminished due to the higher precision in temperature).^32^ This feature makes flow reactors ideal to carry out depolymerisations, as these require high temperatures, precise control and allow for more benign solvents to be used. Further, flow reactors are simple to scale, which makes them highly attractive for industrial application.^32^ Flow reactors also provide conditions of ideal heat transfer, further minimising energy needs. Despite these obvious advantages, to date, the use of continuous flow for depolymerisation reactions has mainly been focussed on depolymerisation of lignin and oligosaccharides, with no reports on the use of flow conditions for CRM of plastics.^33–35^ In this work, with the focus of easier lactide recovery in mind, a series of lower boiling point solvents were explored for PLA depolymerisation in continuous flow (Fig. 1). Various solvents were first tested in batch for this purpose, followed by flow application where temperatures well above the boiling points could be applied with ease. Once the best solvent was identified a range of conditions were explored and kinetic analysis carried out. For this, we made use of inline monitoring of the reaction to provide real-time data on the progress of the reactions, which facilitates the optimisation of the reaction, and which allows for direct insights into the reaction without tedious sampling of aliquots for offline analysis.

## Experimental section

### Materials

All reactions were performed under an atmosphere of argon using standard Schlenk techniques unless otherwise stated. All reagents were purchased from either Sigma Aldrich, Alfa Aesar or Acros Organics and used without further purification unless specified. All solvents used were anhydrous unless otherwise stated. l-LA was recrystallised in dry toluene three times and stored under argon prior to use. 4-Methylbenzyl alcohol was recrystalised in diethyl ether and stored under argon prior to use.

### Instruments

**Fig. 2:**
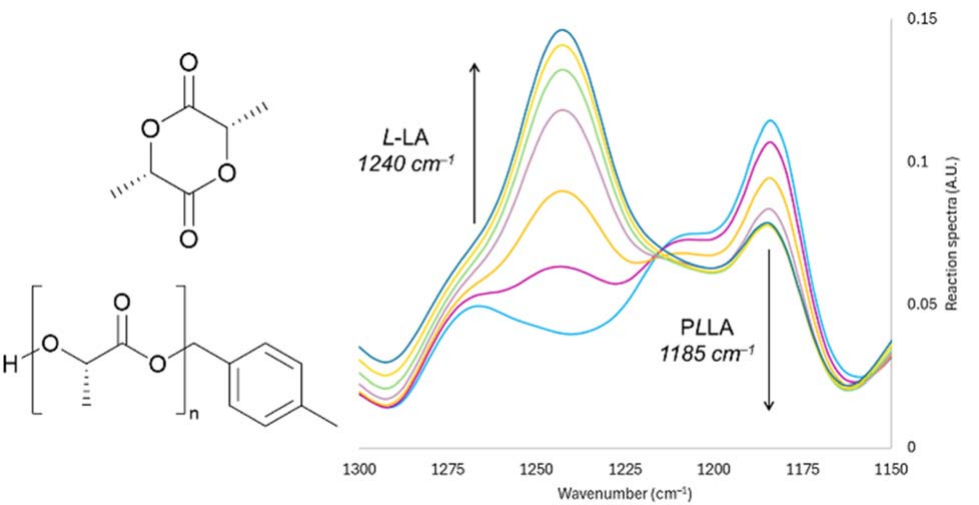
Example of IR absorbances: l-LA (1240 cm^−1^) and PLLA (1185 cm^−1^) in an inline depolymerisation and their structures.

^1^H NMR spectra were recorded on a Bruker 400 MHz instrument and referenced to residual solvent peaks. Coupling constants are given in Hertz. Polymer conversions were determined by ^1^H NMR spectroscopy. Size-exclusion chromatography (SEC) was carried out on a PSS SECcurity2 GPC system operated by PSS WinGPC software, equipped with an SDV 5.0 μm guard column (50 × 8 mm), followed by three SDV analytical 5.0 μm columns with varying porosity (1000 Å, 100 000 Å, and 1 000 000 Å) (50 × 8 mm) and a differential refractive index 3 detectors using THF as the eluent at 40 °C with a flow rate of 1 mL min^−1^. The SEC system was calibrated using linear narrow polystyrene standards from PSS Laboratories ranging from 682 to 2.52 × 10^6^ g mol^−1^. *In situ* IR monitoring of l-LA polymerisations were carried out using a ReactIR 700 (model 701L) with a Micro Flow Cell DS DiComp 50 μL detector. The monitoring software used was iC IR. Kinetic data was extracted from monitoring the asymmetric ester C–O–C stretching vibrations of l-LA at 1240 cm^−1^ and PLLA at 1185 cm^−1^ (Fig. 2).^36^ Peak heights of the absorption spectra were correlated to conversions from samples taken throughout, analysed by ^1^H NMR spectroscopy. Data analysis in Microsoft Excel allowed for the construction of first-order plots, and thus extraction of the initial reaction rate constant. Flow reactions were carried out using a Vapourtec RS-200 system with a high temperature reactor (HTR, Hastelloy C reactor of 2 mL volume).

### Synthesis of PLLA

l-LA (20.0 g, 0.139 mol) was added to a round-bottom flask together with 4-MeBnOH (0.170 g, 0.00139 mol) and Sn(Oct)_2_ (0.563 g, 0.00139 mol) as catalysts. ROP was carried out under an argon atmosphere at 100 °C for 1 h, after which the reaction was quenched by cooling. The polymer was dissolved in dichloromethane (DCM) and precipitated in *n*-hexane, of volume at least 10 times the volume of DCM. The precipitation was repeated four times to remove Sn(Oct)_2_ and unreacted monomers. The purified polymer was dried in a fume hood overnight and thereafter under vacuum (2 days at room temperature). The polymer was stored in a glove box under an argon atmosphere. 90%, *M*_n_ 15.8 kDa, *Ð*_M_ 1.10.

### Depolymerisation in looped flow mode

In a general looped flow reaction, PLLA (0.72 g, 5 mmol) was added to a dried Schlenk flask under an argon atmosphere followed by 10 mL of anhydrous solvent ([PLLA] = 0.5 M), once the polymer was fully dissolved Sn(Oct)_2_ was added (0.18 mL, 10 mol%). Under constant stirring and argon supply, the Schlenk flask was the feed input for the Vapourtec R-200 system equipped with a 2 mL Hastelloy high temperature tube reactor and 250 psi back-pressure regulator at the output. This outlet feed fed back into the starting Schlenk flask to create a closed loop system. An in-line IR system was integrated using a Mettler Toledo ReactIR with a micro flow cell (DiComp), reactions were monitored using the iC IR software. The in-line IR was positioned between the Vapourtec HPLC pump and the high temperature tube reactor. Once the reactor was up to temperature (140–170 °C), the flow rate was set to 1 mL min^−1^, initially the output was set to waste until the PLLA had flushed through the reactor where it was then fed back into the feed solution. Samples were taken intermittently to cross-check IR signals; reactions were run until a steady state was observed. A looped flow approach with a semi-batch reservoir was chosen to allow for flexibility in the study on reaction times. A linear reactor would present considerable limitations with regard to the maximum reaction time accessible. With the looped flow, flow rates can be chosen to be fairly high (reducing resident time distribution issues) while allowing for extended reaction times. Still, the chosen design features all advantages typically associated with flow chemistry, that is flash heating, efficient heat and mass transfer as much as continuous operation. The reactor setup already features the ability to scale the reaction up (by increasing the semi-batch and the continuous flow reactor compartment volume), which can be extended further by feeding PLA into the semi-batch reservoir while removing monomer *via* distillation or other means.

## Results and discussion

### Looped flow depolymerisations

#### Solvent variation

**Fig. 3:**
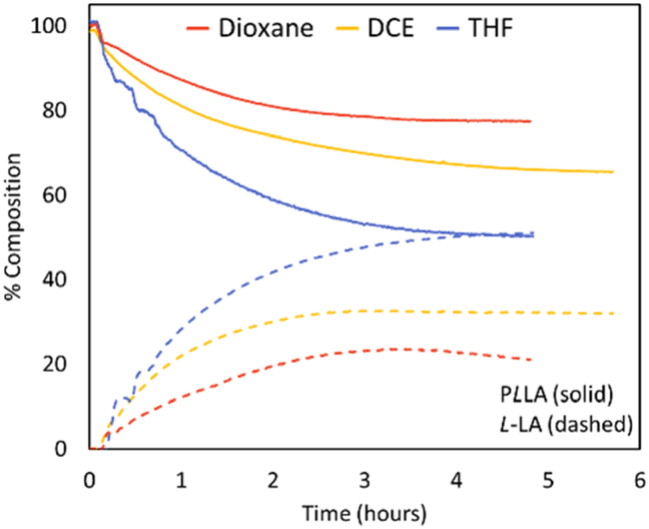
Inline IR traces of PLLA depolymerisation under looped flow conditions ([PLLA] 0.5 mol L^−1^; Sn(Oct)_2_ 10 mol%; 150 °C). PLLA shown in solid lines, l-LA shown in dashed lines. Dioxane (red), DCE (yellow), THF (dark blue).

A range of low boiling point solvents were explored for use in PLA depolymerisation. Due to the closed system of the combined semi-batch-looped flow process, the solvents could be safely superheated (>140 °C) without the need for specialised batch equipment. Further, with the inline IR integrated into the system, real-time monitoring of the reaction allows for greater data output and a visualisation of reaction progress without disturbing the reactions equilibrium. With the reactor setup, a full loop of the reaction medium would take 5.5 minutes, while the residence time in the heated flow reactor compartment was 2 min. Hence, per hour reaction time, depolymerisation would effectively take place for roughly 22 min. As shown in Fig. 3, THF was the most successful solvent under these conditions ([PLLA] 0.5 mol L^−1^, Sn(Oct)_2_ 10 mol%, 150 °C), reaching 51% conversion within 5 hours as compared to other typically used solvents such as dioxane and dichloroethane (DCE). It should be noted that also Me-THF showed promising results (see ESI Section).† While Me-THF would hence be preferrable from a yield and a green solvent perspective, it also unfortunately exhibited significantly lower solubility for PLA and caused PLA to precipitate. Since solubility is a pre-requisite to maintain homogenous reaction conditions and notably to keep pumps operating efficiently, and because differences to pure THF were relatively small, Me-THF was discarded for further experiments.

When depolymerisation was tested under batch conditions, only 3% lactide was achieved ([PLLA] 0.5 mol L^−1^, Sn(Oct)_2_ 10 mol%, 80 °C, 3 h), highlighting the benefits of employing a looped flow system over a traditional batch type. While a direct comparison may appear unfair due to the large temperature difference, it is noteworthy to add that a batch system simply does not allow for more elevated temperatures as solvents would evaporate almost instantaneously. Further, pressurised batch reactors are associated with considerably higher risk profiles. Hence, this comparison presents a classical advantage of using flow reactors, that is the shift of the operation window, which in itself then leads to the process intensification and increase in yield.

The SEC data shows both a reduction in peak intensity and molecular weight of the polymer with a slight increase in *Ð*_M_ as the reaction progressed. When molecular weight is plotted against conversion (Fig. S6†), a linear relationship can be seen; this linear relationship indicates that depolymerisation likely occurs through an unzipping mechanism, where the –OH propagating chain-ends undergo back-biting to reform the cyclic monomer. These results are consistent with another paper which found depolymerisation in solvated conditions to proceed through the same mechanism.^29^

The fraction of *meso*-LA in the lactide mixture was investigated using the CH_3_ signals found in the NMR spectrum. Levels of *meso*-LA formation (Table S2†) followed the same trends seen for rates: THF produced the highest levels of *meso*-LA, reaching a final content of 3.6% (initial *meso*-LA content 0.86%). Conversely, *meso* content found in depolymerisation in DCE showed fluctuations, resulting in a final *meso*-LA content of 1.7%, whereas the use of dioxane showed both low conversions and low *meso*-LA formation. Work by Odelius and co-workers had also previously found solvent choice to have an impact on levels of *meso*-LA formation in batch, where DMSO reduced the selectivity of l-LA formation compared to DMF (*meso*-LA 3.5%, DMF; *meso*-LA 13.2%, DMSO; 140 °C, 0.5 mol L^−1^, Sn(Oct)_2_ 10 mol%).^29^ Nonetheless, despite the higher *meso* content obtained with THF as the reaction solvent, THF is considered a greener option due to its lower toxicity and relatively better environmental impact.^37^ Moreover, through process optimisation it may be possible to control levels of epimerisation seen. THF was therefore selected as solvent for further work.

#### Temperature effects

**Fig. 4:**
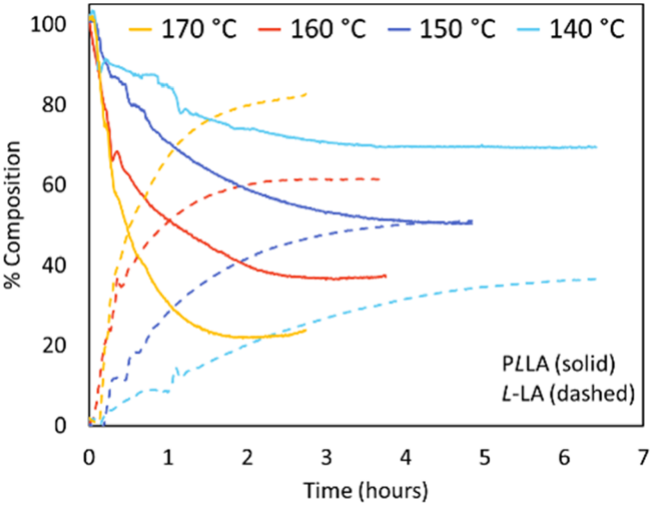
Inline IR traces of PLLA depolymerisation under looped flow conditions ([PLLA] 0.5 mol L^−1^; Sn(Oct)_2_ 10 mol%; THF). PLLA shown in solid lines, l-LA shown in dashed lines. 170 °C (yellow), 160 °C (red), 150 °C (dark blue), 140 °C (blue).

The effect of temperature on depolymerisation rates in THF was then investigated. Temperatures were varied between 140 and 170 °C ([PLLA] 0.5 mol L^−1^; Sn(Oct)_2_ 10 mol%, Fig. 4). As expected for a reaction with both negative Δ*H*_p_ and Δ*S*_p_, the higher temperatures pushed the monomer equilibrium [LA]_eq_ higher (eqn (1)).^17,23^ At 170 °C, 50% of PLLA had been converted to lactide within half an hour, eventually reaching equilibrium at 82% conversion within three hours. In previously published work, a raise in temperature had little effect on the rate of reaction, but significantly increased the number of side reactions; increasing temperature from 140 °C to 180 °C raised the final acrylic acid content from 0.2% to 7.4% (DMSO, 0.5 mol L^−1^, Sn(Oct)_2_ 10 mol%).^29^

Positively, when using THF as the reaction solvent the formation of acrylic acid could not be detected at any temperature. It was found *meso*-LA contents typically increased with the raised reaction temperatures (Table S3†), however, the level of *meso*-LA content remained relatively low, where a maximum final *meso* content of 4.3% was obtained when the reaction temperature was 160 °C at 0.5 mol L^−1^.

#### Concentration effects

**Fig. 5:**
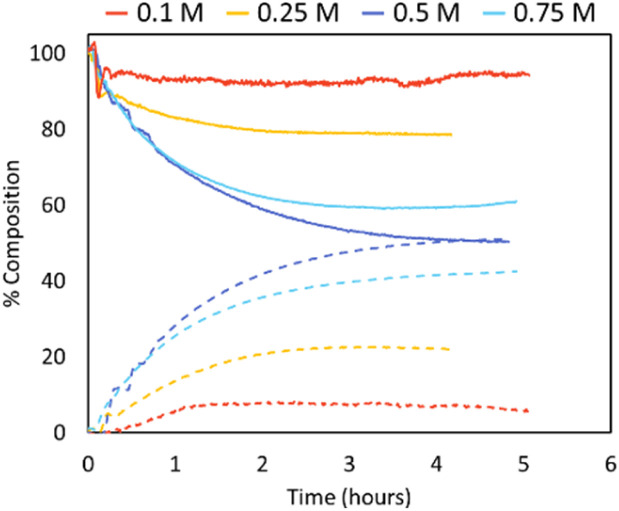
Inline IR traces of PLLA depolymerisation under looped flow conditions (varied concentrations, Sn(Oct)_2_ 10 mol%, THF, 150 °C). PLLA shown in solid lines, l-LA shown in dashed lines. 0.1 M (red), 0.25 M (yellow), 0.5 M (dark blue), 0.75 M (blue).

When exploring different PLLA concentrations, it was noted that when initial polymer concentrations were reduced from 0.5 mol L^−1^ to 0.25 mol L^−1^ or 0.1 mol L^−1^ the extent of depolymerisation decreased (Fig. 5), with depolymerisation conversions not surpassing 22% and 6%, respectively, after 5 hours. However, it was expected that a lower initial concentration would reduce *T*_c_, and therefore increase levels of depolymerisation. While other factors can influence rates of depolymerisation such as chain length, polymer structure, catalyst type and concentrations,^27–29,38–40^ all depolymerisations were carried out on the same batch of PLLA and performed under the same reaction conditions. It was therefore thought these unexpected results were due to the reduced loadings of Sn(Oct)_2_. As catalyst loadings were 10 mol% in relation to PLLA, the reduced polymer concentrations mean longer reaction times would likely be needed to reach equilibrium due to reduced kinetics of the reaction. Previous work found similar trends, where reducing initial PLLA concentration (and therefore catalyst) from 0.5 mol L^−1^ to 0.25 mol L^−1^ dropped PLA conversions from 62% to 44%, respectively (DMSO, 180 °C, Sn(Oct)_2_ 10 mol%, 2 h).^29^ However, within this work, as the inline trace shows a halt in reaction and not just slowed kinetics, it is possible the catalyst is being poisoned over time; an effect which becomes more apparent with the lower catalyst loadings.

**Fig. 6:**
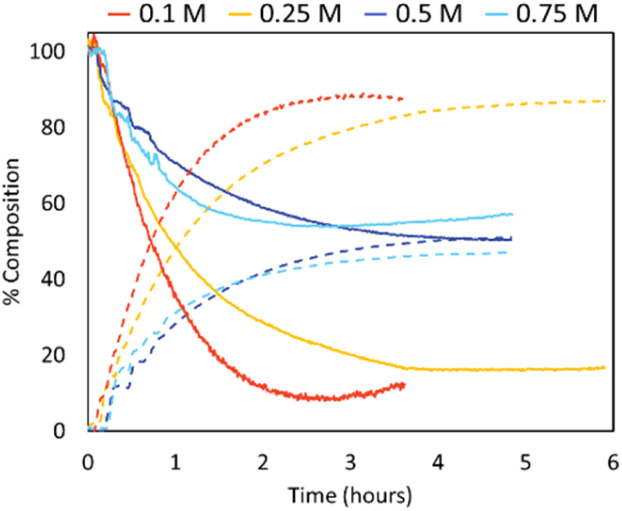
Inline IR traces of PLLA depolymerisation under looped-flow conditions (varied concentrations, Sn(Oct)_2_ 0.06 M, THF, 150 °C). PLLA shown in solid lines, l-LA shown in dashed lines. 0.1 M (red), 0.25 M (yellow), 0.5 M (dark blue), 0.75 M (blue).

Depolymerisations were repeated using the same Sn(Oct)_2_ concentration throughout (0.06 mol L^−1^; Fig. 6). When the catalyst concentration remained constant, the trends with initial polymer concentration became as expected, with lower initial PLA concentrations increasing the extent of depolymerisation. For example, reducing concentration from 0.75 mol L^−1^ to 0.1 mol L^−1^ increased LA production from 47% to 88%, respectively.

#### Arrhenius plot

**Fig. 7:**
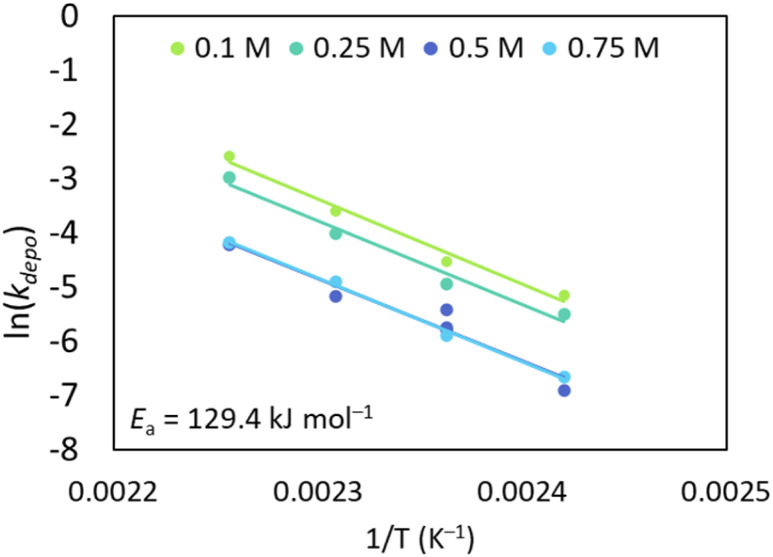
Arrhenius plots of PLLA using 0.06 mol L^−1^ Sn(Oct)_2_. 0.1 M (130.4 kJ mol^−1^, *A* = 1.57 × 10^14^ s^−1^); 0.25 M (*E*_a_ = 128.4 kJ mol^−1^, *A* = 6.04 × 10^13^ s^−1^); 0.5 M (*E*_a_ = 130.2 kJ mol^−1^, *A* = 3.21 × 10^13^ s^−1^); 0.75 M (*E*_a_ = 128.8 kJ mol^−1^, *A* = 2.39 × 10^13^ s^−1^).

To help understand the temperature dependence of PLLA depolymerisation in THF, Arrhenius plots were constructed at four different initial concentrations. It is important to note that an analysis of reaction rates only is meaningful as long as the depolymerisation is far from its equilibrium state (since the net reaction rate will reach zero at equilibrium concentration by definition). Hence, we used only the initial data at the onset of depolymerisation to determine a depolymerisation rate coefficient *k*_depo_. The inline analysis combined with the flash heating capacity of the flow reactor allows for experiments with a meaningful analysis in this case, as no heating up phase needs to be considered that may interfere with the obtained rate information. The temperature dependence of *k*_depo_ at different initial concentrations is depicted in Fig. 7, temperature ranges were studied between 140–170 °C. At each single temperature, almost the same activation energy of *k*_depo_ was identified, with an average of 129.4 kJ mol^−1^. Generally, such high activation energies make sense, given the endothermicity of the depolymerisation reaction, and onset at high *T*_c_. Activation energies were similar to those obtained when studying the effect of tin content in PLLA pyrolysis to a range of products, where an *E*_a_ of 130 kJ mol^−1^ was obtained for a tin content of 60 ppm, studies were conducted in bulk and over a larger temperature frame (40–400 °C).^38^ Another investigated the pyrolysis activation energy of PLLA between 100–400 °C, comparing hydrogen and calcium end-capped polymer, with the calcium end-capped polymer reducing the *E*_a_ from 176 to 98 kJ mol^−1^.^40^ Whereas, when PLA hydrolysis studies have been investigated, activation energies for these reactions are significantly lower (*E*_a_ = 51.0 kJ mol^−1^, 180–250 °C).^41^ To the best of our knowledge, no activation energies have been reported for the depolymerisation of PLA to LA under solvated conditions until now.

## Conclusions

Previous works reported the successful use of high boiling point solvents to lower the *T*_c_ of PLLA, towards an efficient and selective CRM process.^29^ Our work explored the use of a continuous flow system that allowed the use of low-boiling point solvents for PLLA CRM, in order to aid in later monomer recovery. We successfully identified THF as a suitable solvent under a range of temperatures. Depolymerisation conversions reached up to 92% in 1.2 h ([PLLA] = 0.1 mol L^−1^, [Sn(Oct)_2_] = 0.06 mol L^−1^, THF, 170 °C, 5% *meso*-LA). While this is an example where a high catalyst loading was used, depolymerisations under conditions with 10% of catalyst also result in high crude monomer yields, for example 82% in 3 h ([PLLA] = 0.5 mol L^−1^, [Sn(Oct)_2_] = 0.05 mol L^−1^, THF, 170 °C, 3.7% *meso*-LA). While we did not recycle the catalyst in this present work, it is possible to continuously run the reactor without quenching of the reaction, giving rise to potential upscaling of the process. Also the use of immobilised catalysts are in principle possible. Construction of an Arrhenius plot revealed activation energy of the reaction to be 129.4 ± 1.0 kJ mol^−1^, and *meso*-LA content of the recovered monomer was between 3–6% depending upon conditions used. These results showcase the potential of continuous flow processes to facilitate the chemical recycling of PLA back to monomer, under fast rates and mild conditions. Continuous flow chemistry presents itself here as an enabling technology that allows to shift the process window into conditions that are not conventionally accessible to solution-based batch processes. We envision that in future studies it will also be possible to use the same strategy for depolymerisation of other polyesters, for example polycaprolactone.

## Data availability

The data supporting this article have been included as part of the ESI.†

## Author contributions

SE: investigation, data curation, formal analysis, visualisation, writing – original draft. AB and TJ: supervision, conceptualisation, resources, writing – final draft.

## Conflicts of interest

AB has received funding and is collaborating on other topics with TotalEnergies Corbion, a PLA manufacturer.

## Supplementary Material

SC-016-D4SC05891G-s001
