# Supplementary material for: Depolymerisation of poly(lactide) under continuous flow conditions
Source: Chem Sci. 2024 Nov 12;16(1):211–7. doi: 10.1039/d4sc05891g (PMC11587992; doi:10.1039/d4sc05891g)
Supplement: SC-016-D4SC05891G-s001 [file SC-016-D4SC05891G-s001.pdf]

Supplementary Information (SI) for Chemical Science.
This journal is © The Royal Society of Chemistry 2024

Supporting Information

Depolymerisation of Poly(Lactide) Under Continuous Flow Conditions

Sophie Ellis,[a,b] Antoine Buchard[*c] and Tanja Junkers [*a]

[a] Polymer Reaction Design group, School of Chemistry, Monash University, 17 Rainforest Walk, Clayton VIC 3800, Australia. E-mail: tanja.junkers@monash.edu
[b] Department of Chemistry, Institute for Sustainability, University of Bath, Claverton Down, Bath BA2 7AY, U.K.
[c] Department of Chemistry, University of York, York, YO10 5DD, UK. E-mail : antoine.buchard@york.ac.uk..

Table of Contents

1. Experimental details and procedures S2
1.1 Materials S2
1.2 Instruments S2
1.3 Synthesis of P*L*LA S3
1.4 Depolymerization of PLA in batch S3
1.5 Depolymerisation in semi-batch mode S3
2. Experimental data S5
2.1 P*L*LA characterisation data S5
2.2 P*L*LA Depolymerisation data S6
2.2.1 Batch depolymerisation S6
2.2.2 SEC traces and molecular weight monitoring data in continuous flow S7
2.2.3 Calculation of *Meso*-LA content in depolymerisation reactions S8
2.2.4 Inline depolymerisation data in continuous flow S9

1. Experimental details and procedures

1.1 Materials

All manipulations were performed under an atmosphere of argon using standard Schlenk techniques unless otherwise stated. All reagents were purchased from either Sigma Aldrich, Alfa Aesar or Acros Organics and used without further purification unless specified. *L*-LA was recrystallised in dry toluene three times and stored under argon prior to use. 4-Methylbenzyl alcohol was recrystalised in diethyl ether and stored under argon prior to use. All solvents used were anhydrous unless otherwise stated. All other chemicals were purchased from commercial suppliers without further purification unless otherwise stated.

1.2 Instruments

All polymerisations were carried out under an argon atmosphere in the glovebox or using standard Schlenk line techniques. ^1H NMR spectra were recorded on a Bruker 400 MHz instrument and referenced to residual solvent peaks. Coupling constants are given in Hertz. Polymer conversions were determined by ^1H NMR spectroscopy. Size-exclusion chromatography (SEC) was carried out on a PSS SECcurity2 GPC systems operated by PSS WinGPC software, equipped with an SDV 5.0 µm guard column (50 x 8 mm), followed by three SDV analytical 5.0 µm columns with varying porosity (1000 Å, 100000 Å, and 1000000 Å) (50 x 8 mm) and a differential refractive index 3 detectors using THF as the eluent at 40 °C with a flow rate of 1 mL min^{-1}. The GPC system was calibrated using linear narrow polystyrene standards from PSS Laboratories ranging from 682 to 2.52 x 106 g mol^{-1}. *In-situ* ReactIR monitoring of *L*-LA polymerisations were carried out using a ReactIR 700 (model 701L) with a Micro Flow Cell DS DiComp 50 µL detector. Monitoring software used for both was iC IR. Kinetic data was extracted from monitoring the C-O-C stretching mode of *L*-LA 1240 cm^{-1} and P*L*LA 1185 cm^{-1}.[2] Peak heights of the absorption spectra were correlated to the final conversion of polymer, analysed through ^1H NMR spectroscopy. Data analysis in Microsoft Excel allowed for the construction of first-order plots, and thus extraction of the initial reaction rate constant.

1.3 Synthesis of P*L*LA

L-LA (20.0 g, 0.139 mol) was added to a round-bottom flask together with 4-MeBnOH (0.170 g, 0.00139 mol) and $Sn(Oct)_2$ (0.563 g, 0.00139 mol) as catalysts. ROP was carried out under an argon atmosphere at 100 °C for 1 hr, after which the reaction was quenched by cooling. The polymer was dissolved in dichloromethane (DCM) and precipitated in n-hexane, of volume at least 10 times the volume of DCM. The precipitation was repeated four times to remove $Sn(Oct)_2$ and unreacted monomers. The purified polymer was dried in a fume hood overnight and thereafter under vacuum (2 days at room temperature). The polymer was stored in a glove box under an argon atmosphere. 90%, M_n 15.8 kDa, $Đ_M$ 1.10.

1.4 Depolymerization of PLA in batch

The solvent, polymer concentration, catalyst, catalyst concentration and temperature were varied to explore the effect on the depolymerization, in batch and semi-batch conditions. In a general batch reaction, P*L*LA (0.36 g, 2.5 mmol) was added to a 10 mL Schlenk flask equipped with a magnetic stirrer together with catalyst (5-10 mol %; $Sn(Oct)_2$) and 5.0 mL of solvent (THF, DCE, or ACN), resulting in a concentration of 0.5 M (based on the *L*-LA repeating unit). The preparation was performed inside a glove box under an argon atmosphere. The flask was placed in an oil bath (80 °C). Aliquots of 0.5 mL were taken at regular time intervals, quenched by cooling. 0.1 mL was added to 0.5 mL of $CDCl_3$ spiked with 0.1 M benzoic acid and analysed by ^1H NMR spectroscopy without further purification, remaining 0.4 mL has solvent removed and added to 1.5 mL THF for GPC analysis.

1.5 Depolymerisation in semi-batch mode

In a general flow reaction, P*L*LA (0.72 g, 5 mmol) was added to a dried Schlenk flask under and argon atmosphere followed by 10 mL of anhydrous solvent ([P*L*LA] = 0.5 mol L^{-1}, based on the *L*-LA repeat unit), once the polymer was fully dissolved $Sn(Oct)_2$ was added (0.18 mL, 10 mol %, 0.06 mol L^{-1}). Under constant stirring and argon supply, the Schlenk flask was the feed input for the Vapourtec R-200 system (**Figure S2**), equipped with a 2 mL Hastelloy high temperature tube reactor and 250 psi back-pressure regulator at the output. This outlet feed fed back into the starting Schlenk flask to create a closed loop system. An in-line IR system

was integrated using a Mettler Toledo ReactIR with a micro flow cell (DiComp), reactions were monitored using the iC IR software. The in-line IR was positioned between the Vapourtec HPLC pump and the high temperature tube reactor. Once the reactor was up to temperature (140-170 °C), the flow rate was set to 1 mL/min, initially the output was set to waste until the P*L*LA had flushed through the reactor where it was then fed back into the feed solution. Samples were taken intermittently to cross-check IR signals: aliquots of 0.5 mL were taken at regular time intervals, quenched by cooling, 0.1 mL was added to 0.5 mL of $CDCl_3$ spiked with 0.1 M benzoic acid and analysed by ^{1}H NMR spectroscopy without further purification, remaining 0.4 mL has solvent removed and added to 1.5 mL THF for GPC analysis. Reactions were run until a steady state was observed. IR bands used for monitoring reaction progress were the C-O-C stretching bands found at 1240 cm^{-1} (*L*-LA) and 1185 cm^{-1} (P*L*LA).

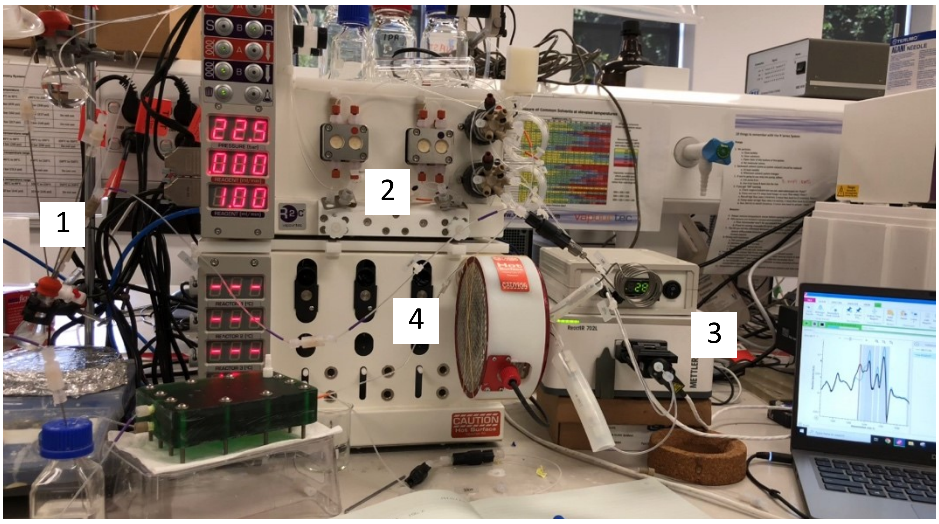

Figure S1 *Semi-batch Vapourtec set up with inline IR monitoring. Schlenk flask for input/output feed (1), Vapourtec pump system (2), inline IR system (3), high temperature reactor (4).*

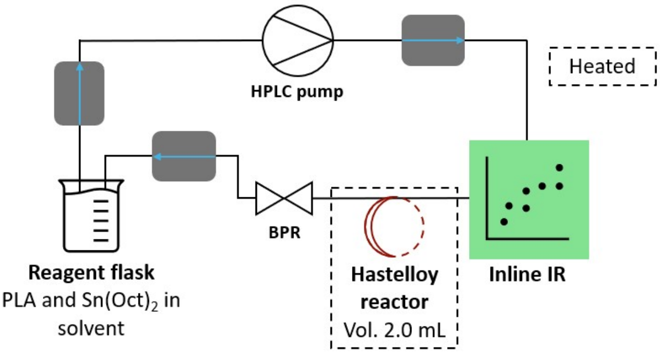

Figure S2 *Continuous flow depolymerisations setup with inline ReactIR analysis. BPR (back-pressure regulator) = 250 psi.*

2. Experimental data

2.1 PLLA characterisation data

Table S1 Synthesis of PLLA for depolymerisation using Sn(Oct)2 catalyst, loadings of [LA]:[Cat]:[I] = 100:1:1, in melt at 100 °C.

Entry	Time (h)	Conv.[a] (%)	$M_{n,\ Theo}$	$M_{n,\ NMR}$	$M_{n,\ SEC}$	$Đ_M$
1	1	90	13 000	15 250	15 800	1.10

[a]Determined from the ^{1}H NMR spectrum. [b]Theoretical Mn = (144.13 x equiv. LA) x (conv./100). [c]As determined by SEC (THF) using RI methods, relative to poly(styrene) standards (multiplied by a factor of 0.58, rounded to the nearest 50).

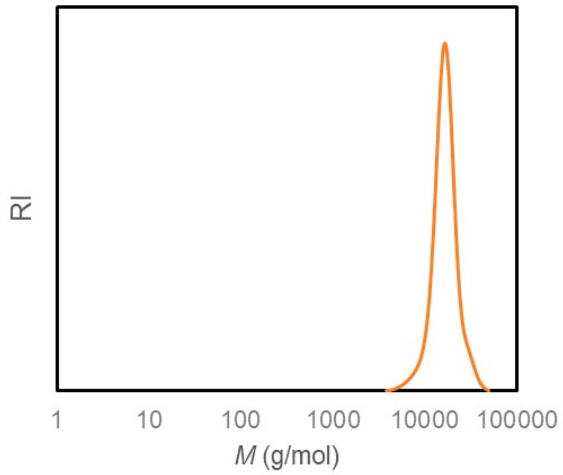

Figure S3 SEC trace of synthesised PLLA for depolymerisation.

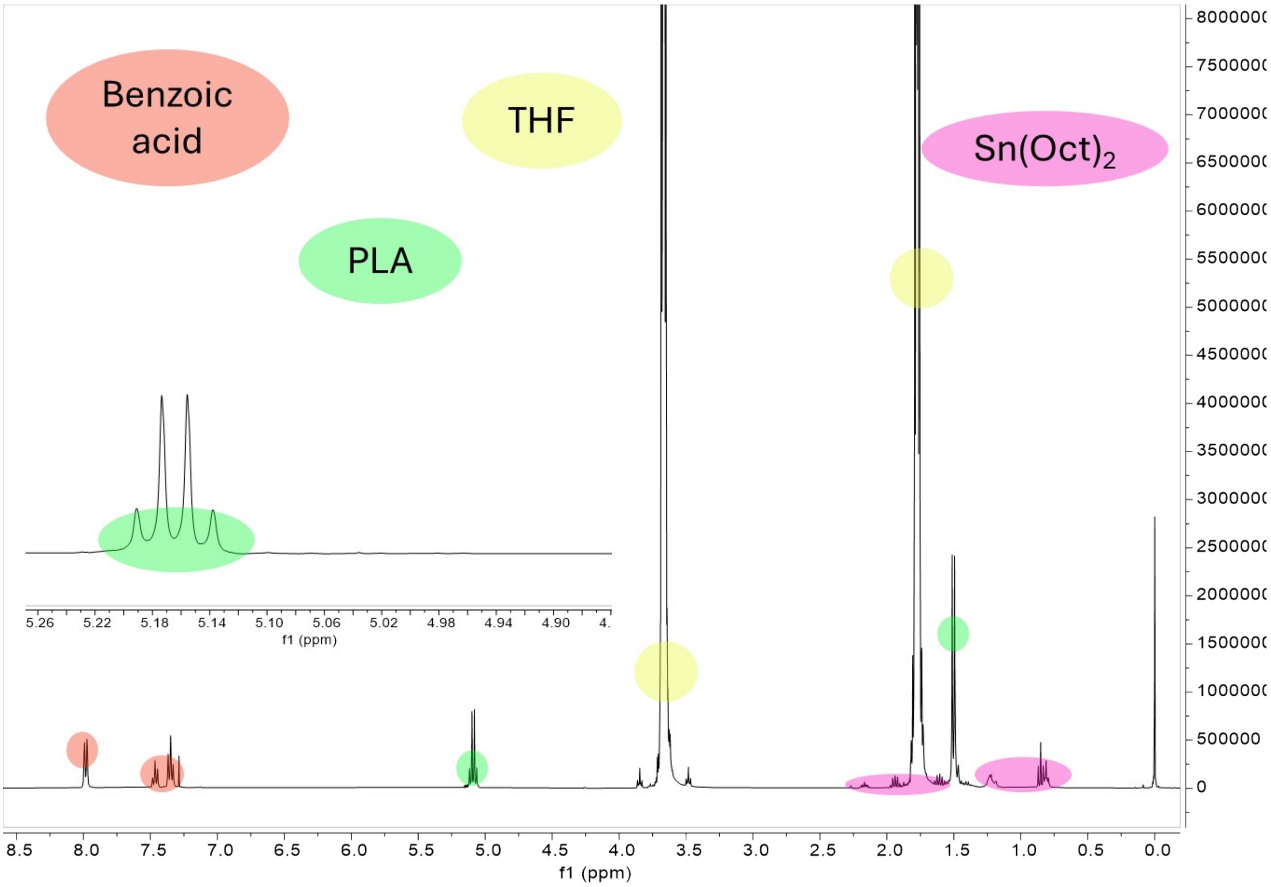

Figure S4 ¹H NMR spectroscopic assignment of sample taken from PLLA depolymerisation in THF, initial sample prior to depolymerisation.

2.2 P*L*LA Depolymerisation data

2.2.1 Batch depolymerisation

Initial experiments used batch conditions to explore the suitability of various low boiling point solvents (<~100 °C) for depolymerisation of P*L*LA. The depolymerisation temperature was set at 80 °C for all solvents in this initial screening; whilst this temperature is too low for sufficient depolymerisation to occur, the maximum temperature was limited due to batch conditions, the obtained results were intended for screening purposes only. Solvents were selected on the ability to dissolve PLA (relevant for continuous flow conditions) fully or partially and having a boiling point low enough for easy lactide recovery. Samples were collected and analysed by NMR spectroscopy at set time intervals between 0 and 3 hours. Results in Figure S5 show acetonitrile (ACN) to be the least suitable solvent, with only trace amounts (<1%) of *L*-LA being produced within the three-hour reaction time, whereas the green solvent 2-methyl tetrahydrofuran (2-MeTHF) showed the highest potential reaching 5% conversion. Other

solvents tested were dioxane, dichloroethane (DCE), and tetrahydrofuran (THF) which all showed similar results. The scattered data collected <60 minutes is due to the large signal to noise ratio found in NMR spectroscopy at lower conversions. No GPC data was collected due to the low conversions. Whilst low conversions were reached under batch conditions, all solvents bar ACN show potential in depolymerisation, and so were selected for further study.

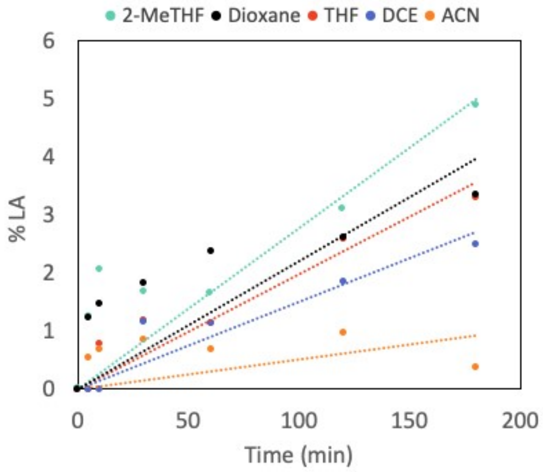

Figure S5 Batch depolymerisation results for PLLA in various solvents at 80 °C using Sn(Oct)$_2$ ([PLLA] 0.5 M; catalyst 10 mol %).

2.2.2 SEC traces and molecular weight monitoring data in continuous flow

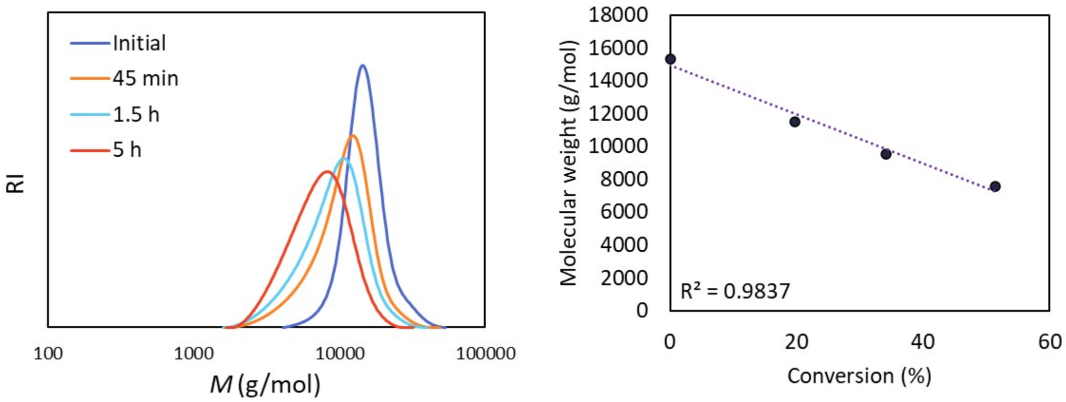

Figure S6 *SEC traces of depolymerisation carried out in THF ([PLLA] 0.5 M; Sn(Oct)$_2$ 10 mol %; 150 °C)* ***(LHS)****. Decrease in molecular weight in relation to polymer conversion from same data set, molecular weight determined by GPC analysis* ***(RHS)****.*

2.2.3 Calculation of *Meso*-LA content in depolymerisation reactions

Calculations of *meso*-, *L*-, and *D*- contents were based on previously published works.[1, 2]

The fraction of *meso*-LA was calculated from the integrals of the CH_3 peaks for *meso*-LA and *L*-LA/*D*-LA:

$$m = \frac{\int CH_3(meso-LA)}{\int CH_3(meso-LA) + \int CH_3(L-LA/D-LA)}$$

$$L = \frac{((1+\sqrt{1-2m})^2}{4}$$

$$D = \frac{((1-\sqrt{1-2m})^2}{4}$$

Total conversion was calculated from the CH signals in NMR spectra, and relative amounts of *L*-LA, *D*-LA, and *meso*-LA is calculated as follows:

L-LA % = L ∗ conversion ∗ 100%

D-LA % = D ∗ conversion ∗ 100%

meso-LA % = m ∗ conversion ∗ 100%

P*L*LA % = (1 – C) * 100%

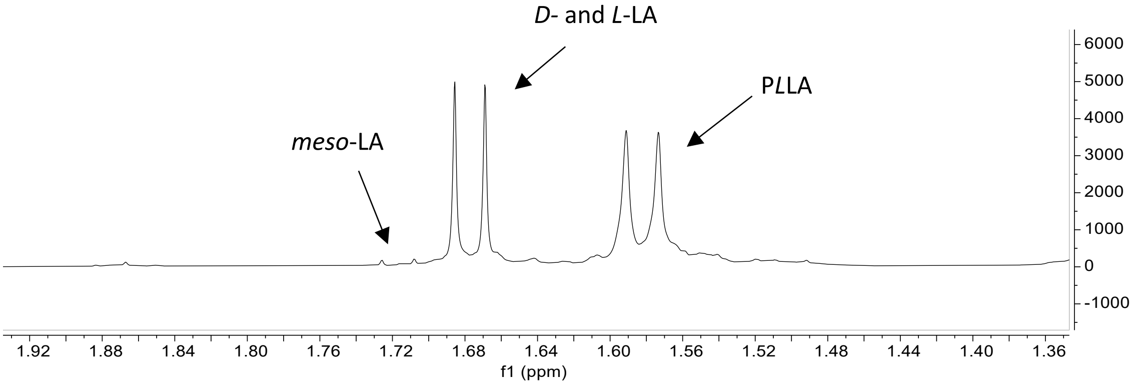

Figure S7 Comparison of CH_3 peaks of meso-LA (1.71 ppm), D-LA and L-LA (1.68 ppm), and PLLA (1.58 ppm) found in NMR spectrum after depolymerisation in THF (0.5 M) at 150 °C.

2.2.4 Inline depolymerisation data in continuous flow

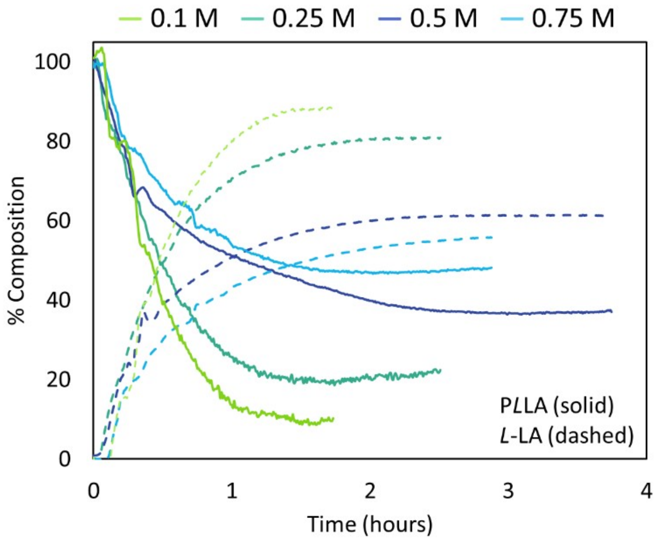

3. *Figure S8 Inline IR traces of PLLA depolymerisation under semi-batch conditions (varied initial concentrations of PLLA, $Sn(Oct)_2$ 0.06 M, THF, 160 °C). PLLA shown in solid lines, L-LA shown in dashed lines. 0.1 M (light green), 0.25 M (dark green), 0.5 M (dark blue), 0.75 M (blue).*

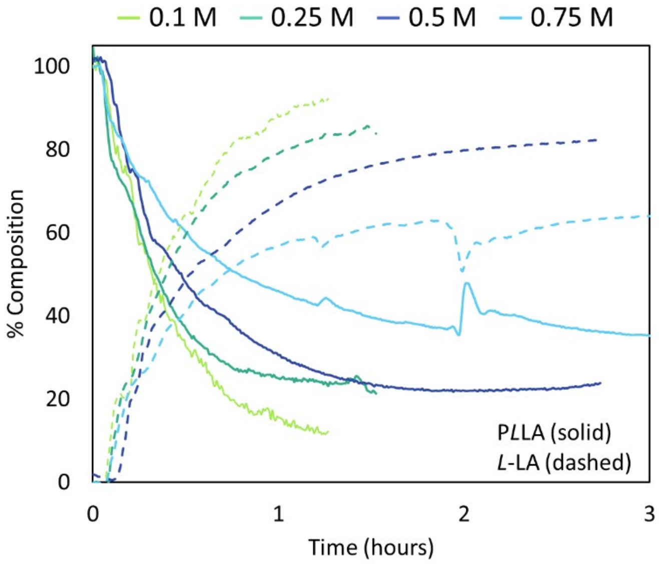

4.

5. *Figure S9 Inline IR traces of PLLA depolymerisation under semi-batch conditions (varied initial concentrations of PLLA, $Sn(Oct)_2$ 0.06 M, THF, 170 °C). PLLA shown in solid lines, L-LA shown in dashed lines. 0.1 M (light green), 0.25 M (dark green), 0.5 M (dark blue), 0.75 M (blue).*

Table S2 Relative amounts of L-LA, D-LA, and meso-LA in varied solvents ([PLLA] = 0.5 mol L^{-1}, $Sn(Oct)_2$ 10 mol%, 150 °C), determined from NMR spectroscopic analysis of samples taken throughout the experiment.

Solvent	Time (h)	*L*-LA (%)[a]	*D*-LA (%)[a]	*meso*-LA (%)[b]	PLA (%)	Total conv. (%)
THF	0.7	19.2	<0.0	0.7	80.0	20.0
	1.5	32.1	0.2	2.0	65.9	34.1
	4.8	48.2	0.5	3.6	48.2	51.8
DCE	0.6	13.7	<0.0	0.5	85.5	14.2
	2	27.1	0.4	2.4	70.5	29.5
	5.7	30.8	0.1	1.7	67.5	32.5
Dioxane	1.1	12.6	<0.0	0.6	86.8	13.2
	2.9	20.8	<0.0	0.7	78.6	21.5
	4.8	23.0	<0.0	0.7	76.4	23.6

[a]Calculated based on probability law. [b]Calculated from integrals of CH_3 peaks of meso-LA and D-/L-LA.

Table S3 Relative amounts of L-LA, D-LA, and meso-LA at varied temperatures and initial PLLA concentrations ($Sn(Oct)_2$ 0.06 mol L^{-1}, THF) determined from NMR spectroscopic analysis of samples taken throughout the experiment.

Conc. [P*L*LA] (mol L^{-1})	Temp. (°C)	Time (h)[a]	*L*-LA (%)[b]	*D*-LA (%)[b]	*meso*-LA (%)[c]	PLA (%)	Total conv. (%)
0.5	140	1.3	15.3	<0.0	0.8	83.9	16.1
		2	21.6	<0.0	0.8	77.6	22.4
		6.3	34.6	<0.0	1.1	64.3	35.8
	150	0.7	19.2	<0.0	0.7	80.0	20.0
		1.5	32.1	0.2	2.0	65.9	34.1
		4.8	48.2	0.5	3.6	48.2	51.8
	160	0.8	35.5	0.2	2.3	62.2	37.8
		2.5	57.0	0.5	4.1	38.8	61.2
		4.1	57.5	0.5	4.3	38.2	61.8
	170	0.3	31.6	0.3	2.2	66.2	33.8
		0.6	62.5	0.4	3.4	48.4	51.6
		2.7	78.3	0.2	3.7	18.0	82.0
0.1	140	1.1	18.8	1.7	3.0	78.1	21.9
		1.5	20.9	3.7	4.3	74.6	25.4
		3.3	29.5	1.5	3.9	66.5	33.5
	150	0.5	31.0	0.4	2.5	66.4	33.6
		1.6	71.2	0.8	5.6	23.1	76.9
		3.1	83.8	0.7	5.9	10.2	89.8
	160	0.7	39.5	0.4	3.1	57.3	42.7
		1.2	57.4	0.2	3.3	39.3	60.7
		1.7	83.8	0.3	4.3	11.8	88.2
	170	0.3	63.3	0.4	3.9	32.7	67.3
		0.5	82.8	0.2	4.0	13.2	86.8
		1.2	87.0	0.4	5.1	7.8	92.2
0.25	140	1	12.0	0.4	1.4	86.5	13.5

		2.3	23.9	0.2	1.8	74.3	25.7
		4.8	29.1	0.3	2.2	68.6	31.4
	150	0.5	26.2	0.3	2.2	71.6	28.4
		1.3	54.1	0.3	3.2	42.6	57.4
		2.6	72.1	0.4	4.3	23.6	76.4
		3.6	78.8	0.5	5.1	16.1	83.9
	160	0.3	32.9	0.3	2.5	64.6	35.5
		1.3	69.8	0.4	4.4	25.7	74.3
		2.5	77.0	0.8	5.8	17.1	83.0
	170	0.2	21.0	0.1	1.1	77.8	22.2
		0.4	52.3	0.2	2.8	45.0	55.0
		1.1	77.9	0.1	3.3	18.9	81.2
		1.5	81.8	0.4	4.6	13.5	86.5
0.75	140	0.7	6.9	0.6	1.1	91.9	8.1
		2.7	14.6	0.7	1.9	83.4	16.6
		4.1	15.7	1.4	2.5	81.7	18.3
	150	0.6	21.7	0.2	1.5	76.8	23.2
		2.2	39.8	0.3	2.8	57.3	42.7
		3.9	43.1	0.3	3.0	53.9	46.1
	160	0.7	33.0	0.1	1.9	65.1	35.0
		1.6	47.0	0.2	2.8	50.1	49.9
		2.6	51.3	0.3	3.3	45.4	54.6
	170	0.5	37.0	<0.0	1.3	61.7	38.3
		1.1	55.8	0.1	2.1	42.1	58.0
		3	62.9	0.1	2.2	34.9	65.1

[a]Rounded to the nearest 0.5 h. [b]Calculated based on probability law. [c]Calculated from integrals of CH_3 peaks of meso-LA and D-/L-LA.

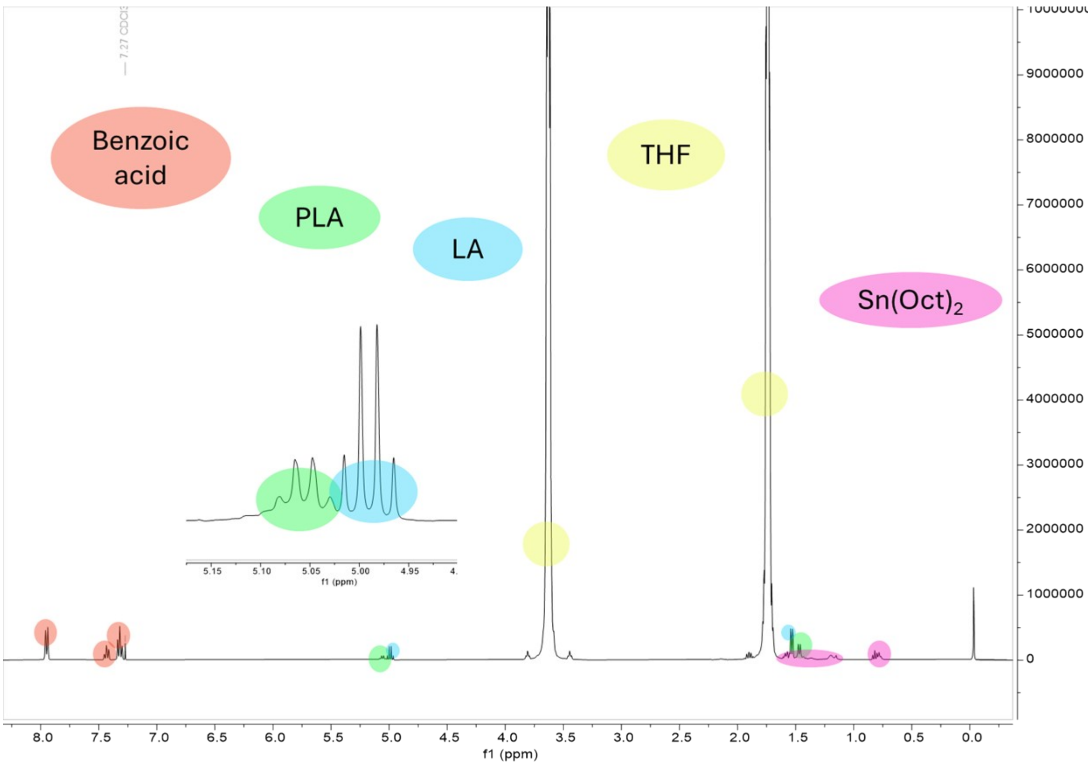

Figure S10 ^{1}H NMR spectrum assignment of sample taken from PLLA depolymerisation in THF at 160 °C after 4 hours (0.5 mol L^{-1}, Sn(Oct)2 0.06 mol L^{-1}).
